# Complete Genome Sequences of Three Isolates of *Xanthomonas fragariae*, the Bacterium Responsible for Angular Leaf Spots on Strawberry Plants

**DOI:** 10.1128/genomeA.00642-17

**Published:** 2017-08-10

**Authors:** Michael Gétaz, Jan M. van der Wolf, Jochen Blom, Joël F. Pothier

**Affiliations:** a Environmental Genomics and Systems Biology Research Group, Institute for Natural Resource Sciences, Zurich University of Applied Sciences (ZHAW), Wädenswil, Switzerland; b Wageningen Plant Research, Wageningen, The Netherlands; c Bioinformatics and Systems Biology, Justus-Liebig University Giessen, Giessen, Germany

## Abstract

*Xanthomonas fragariae* is a worldwide-spread plant bacterial disease causing angular leaf spots, thus reducing the yield of production for strawberry fruits. Three isolates with various geographic and time origins were sequenced with long-read technology (PacBio) to generate finished genome sequences of virulent strains and observe the variability in their contents.

## GENOME ANNOUNCEMENT

*Xanthomonas fragariae* is a bacterium under quarantine status in Europe (1) and causes angular leaf spots of strawberry. The first symptoms were observed and described in the United States in 1960 (2), and further spread took place worldwide. A first *X. fragariae* genome (LMG 25863) was published in 2013 (3), with a draft status of 96 contigs. Recently, two new complete genome sequences of strains (FaP21 and FaP29) originating from California (USA) were published and highlighted the presence of plasmids (4). In this study, three additional strains with different geographic and time origins were sequenced with long-read sequencing technology (PacBio). The strain PD 885, also known as LMG 708, is the type strain, isolated in the United States by B. W. Kennedy in 1962 from *Fragaria chiloensis* var. *ananassa* (2). Strains NBC 2815 and PD 5202 were both isolated in The Netherlands from *Fragaria* × *ananassa* in 2011 and 2005, respectively.

Total genomic DNA was isolated, according to the method of Pitcher et al. (5), from cells grown in liquid Wilbrink-N (6). Library preparation and PacBio sequencing were performed at the Functional Genomics Center Zurich (Zurich, Switzerland) on a PacBio RSII platform using one single-molecule real-time (SMRT) cell per library with 20-kb inserts. Raw data were then assembled with the SMRT Analysis software version 2.3.0 using the HGAP approach (7) and yielded 3 contigs for PD 885^T^ and PD 5205 but only 2 contigs for NBC 2815. After manual inspection using the Lasergene genomics package version 12.1.0 (DNAStar, Madison, WI), all these contigs were found to be circular. To control the assembly and correct possible misassigned bases, raw reads were mapped against the manually edited HGAP assemblies with the SMRT Analysis software version 2.3.0 using the RS_Bridge Mapper protocol. The final assemblies were similar in size (4.2721, 4.2839, and 4.2682 Mbp for PD 885^T^, PD 5205, and NBC 2815, respectively) and in the same range as those previously described (3, 4). The G+C contents were similar in all genomes (62.21, 62.30, and 62.31%) and in a similar range for plasmids (57.77% to 62.96%). Genomes were annotated automatically using GenDB (8), which yielded for PD 885^T^, PD 5205, and NBC 2815 a total of 4,015, 3,994, and 3,939 genes, respectively.

The sequenced strains harbored plasmids with the same backbones as the two plasmids described by Henry and Leveau (4), approximating 29 kb and 21 kb, with low variabilities in plasmid content and lengths between strains. PD 885^T^ harbors two plasmids (29.234 and 27.107 kb), and PD 5205 also contains two plasmids (30.469 and 20.830 kb), whereas only one plasmid (21.045 kb) was detected in NBC 2815. A 10-kb insertion was observed in the 27.107-kb plasmid harbored by PD 885^T^ compared to other 21-kb plasmids. This insertion contains genes encoding a type IV secretion protein, VirB5, and VirB6, which are absent from all other *Xanthomonas fragariae* replicons and chromosomes sequenced so far. A 1-kb insertion containing two genes coding for transposase proteins was detected in the plasmid PD 5205 (30.469 kb) compared to other 29-kb plasmids.

### Accession number(s).

The complete genome sequences for PD 885^T^, NBC 2815, and PD 5202 have been deposited at DDBJ/ENA/GenBank under the accession numbers LT853882 (PD 885^T^ chromosome), LT853883 (plasmid pPD885-29), LT853884 (plasmid pPD885-27), LT853885 (PD 5205 chromosome), LT853886 (plasmid pPD5205-30), LT853887 (plasmid pPD5205-21), LT853880 (NBC 2815 chromosome), and LT853881 (plasmid pNBC2815-21).
